# Endovascular treatment of delayed cerebral ischemia after aneurysmal subarachnoid hemorrhage – an international survey

**DOI:** 10.1186/s12883-025-04480-2

**Published:** 2025-10-31

**Authors:** Michael Veldeman, Thanh N. Nguyen, Johanna Ospel, Charlotte S. Weyland, EYMINT survey and research committee

**Affiliations:** 1 Department of Neurosurgery, RWTH Aachen University Hospital, Aachen, Germany; 2 Radiology, Boston Medical Center, Boston University Chobanian and Avedisian School of Medicine, Boston, MA 02118 USA; 3 Neurology, Boston Medical Center, Boston University Chobanian and Avedisian School of Medicine, Boston, USA; 4 Calgary Stroke Program, Departments of Clinical Neurosciences and Community Health Sciences, The Hotchkiss Brain Institute and the O’Brien Institute for Public Health University of Calgary, Calgary, Canada; 5 Department of Diagnostic and Interventional Neuroradiology, RWTH University Hospital Aachen, Aachen, Germany

**Keywords:** Aneurysmal subarachnoid hemorrhage, Intra-arterial spasmolysis, Angioplasty, Delayed cerebral ischemia, Endovascular treatment, Cerebral vasospasm

## Abstract

**Background:**

Delayed cerebral ischemia (DCI) is a major cause of morbidity after aneurysmal subarachnoid hemorrhage (SAH). Endovascular treatment (ET) has emerged as a rescue strategy, but its optimal timing, indication, and modality remain unclear. This study assessed international ET practices, focusing on treatment variability and clinical decision-making.

**Methods:**

A 25-question survey was developed with input from specialists in interventional neuroradiology, neurosurgery, neurology, and neurocritical care. It was disseminated via professional societies to physicians involved in bedside decisions. Respondents reviewed clinical scenarios representing common DCI presentations, including proximal/distal vasospasm and conscious/unconscious patients. Descriptive analysis was performed.

**Results:**

179 respondents from 38 countries participated; 76.5% reported ET availability at their institution. The most common strategy was single or repeated intra-arterial spasmolysis (76.5%), followed by continuous intra-arterial vasodilator infusion (23.0%). In unconscious patients, 50% applied spasmolysis as first-line treatment. For refractory proximal vasospasm, a stepwise approach was preferred, starting with intra-arterial pharmacologic spasmolysis, then angioplasty. While angioplasty was widely used, 66.5% considered it riskier than spasmolysis.

**Conclusion:**

This survey highlights marked variability in ET practices for DCI. Intra-arterial spasmolysis is the predominant strategy, with alternative approaches like continuous infusion and angioplasty also in use. These findings underscore the need for randomized trials to define optimal ET strategies and inform evidence-based protocols for DCI following SAH.

**Supplementary Information:**

The online version contains supplementary material available at 10.1186/s12883-025-04480-2.

## Background

Delayed cerebral ischemia (DCI) is, after initial hemorrhage severity, the most relevant determinant of functional outcome after aneurysmal subarachnoid hemorrhage (SAH) [1]. Despite advancements in neurocritical care, treatment options for DCI remain limited. Oral nimodipine remains to date, the only prophylactic strategy supported by level one evidence, improving clinical outcome by reducing the risk of DCI evolving into DCI-related infarction [2, 3]. Translational and clinical research have improved our understanding of DCI and focus has shifted away from angiographic vasospasm as the sole culprit. Treatment strategies to reduce angiographic vasospasm (i.e., clazosentan, [4, 5]), failed to improve clinical outcome whereas, oral nimodipine does so without a direct effect on vasospasm. Microvascular dysfunction leading to vasospasm and/or thrombosis on a capillary level, cortical spreading depolarization, autoregulatory disturbance, blood–brain-barrier disruption and neuroinflammation have all been identified as contributors to the pathophysiology of DCI.

Nonetheless, endovascular treatment (ET) of angiographic vasospasm has yielded observational success [6]. Intra-arterial pharmacological spasmolysis is mainly used to treat diffuse and distal vasospasm, whereas transluminal balloon angioplasty is most commonly applied in proximal vasospasm [7–9]. The effects of pharmacological spasmolysis are usually transient as the drug washes out and multiple treatment sessions are often necessary, requiring repeated procedures in critically ill patients. Alternatively, the vasodilatory agent can be continuously delivered intravenously or via an indwelling catheter in either (uni- or bilaterally) internal carotid arteries and/or vertebral arteries. [7] To date two randomized controlled trials (RCTs) assessing the effectiveness of endovascular DCI (in one trial defined as MRI perfusion deficits) treatment have been conducted yielding mixed results. [10, 11] A study applying ET as a first tier therapeutic step, was halted prematurely as clinical outcome proved worse in the endovascular treatment group [10]. The authors concluded that better selection of DCI patients who could benefit from ET is necessary, especially weighing the potential complications of this treatment [12]. In a second trial, good short-term clinical responses were observed in patients who could be examined neurologically [11]. The possibilities of ET for the treatment of DCI and vasospasm after SAH might widen by the emerging use of temporary stent retriever deployment [13].

An RCT investigating ET as a rescue treatment, as it is most commonly used in current clinical practice, has not been performed [14, 15]. A better understanding of ET practice patterns is required to design such a trial in a manner that patient selection, the trial workflow, treatment algorithm, and clinical endpoints are accepted and adopted by the participating study centers. There have been a few surveys assessing differences in endovascular DCI and vasospasm management [16, 17]. These surveys documented a high heterogeneity in the diagnostic and therapeutic management of DCI. However, they did not evaluate the use of continuous pharmacological spasmolysis.

### Objectives

This survey-based study aims to explore endovascular treatment practice patterns for DCI in detail. This includes also the reasons why it might not be employed in some institutions. This information is intended to aid in the design of a treatment algorithm which would be easily adopted by centers already applying endovascular treatment for a future planned RCT.

## Methods

### Survey design and distribution

A 25-question survey was developed in collaboration with specialists in interventional neuroradiology, neurosurgery, neurology, and neurocritical care. To get a broader picture of incentives beyond endovascular technicalities, neurointensivist were included as part of the target group. Personal from centers not providing ET was not discouraged from partaking. In this case the questionnaire collapsed and only questions related to the reasons why the treatment was not offered were presented. The survey was accessible online via the Survey Planet platform from January 6 to May 15, 2025. To optimize response rates, the questionnaire was concise and straightforward, with an estimated completion time of 10–15 min. The survey was distributed through targeted outreach to practicing physician members of professional societies, including but not exclusively the European Society of Minimally Invasive Neurological Therapy (ESMINT), the Society of Vascular and Interventional Neurology (SVIN), and the Society of Neurointerventional Surgery (SNIS), via email invitations and member page postings. Additionally, the survey link was disseminated electronically to members of the German Society of Neuroradiology (DGNR). The full questionnaire and results are provided as Supplemental Material (Appendix 1).

### Study population and participant selection

The survey targeted medical professionals actively involved in bedside decision-making for DCI treatment in SAH. It aimed to capture institutional treatment approaches by recruiting one representative per center. Internet protocol addresses were saved anonymously by the survey’s online platform and manually screened to prevent duplicate response bias.

### Ethical considerations

The study was approved by the local ethics committee of the medical faculty of RWTH Aachen University (Institutional Review Board, reference number EK 25/103). In accordance with national regulations (§15, Berufsordnung für die Ärztinnen und Ärzte in Nordrhein) and the decision of the ethics committee, the requirement for written informed consent was waived, as participation in the survey was voluntary, anonymous, and involved no personal or sensitive health information. All responses were anonymous, and no financial or other incentives were provided. Data collection and reporting adhered to the Consensus-Based Checklist for Reporting of Survey Studies (CROSS) guidelines [18].

### Survey content and case scenarios

The survey included four clinical scenarios designed to assess decision-making in different presentations of DCI. The cases were developed to explore treatment thresholds and preferred therapeutic strategies under varying clinical and radiological conditions. In the survey, we defined spasmolysis as endovascular treatment via intra-arterial application of a vasodilator agent i.e., pharmacological spasmolysis.

#### Case 1 – ET as First- or second-line treatment (Conscious Patient)

A patient with first onset of clinical DCI presents with a perfusion deficit on computed tomography perfusion (CTP) imaging. Angiographic vasospasm is present in the proximal cerebral arteries, potentially amenable to both spasmolysis and angioplasty. The primary objective was to assess the propensity to initiate ET *versus* attempting induced hypertension first.

#### Case 2 – ET as First- or second-line treatment (Unconscious Patient)

Case 2 includes the same clinical and radiological scenario as Case 1, but with the patient now in an unresponsive state, rendering neurological examination unfeasible. The case was designed to gauge whether the inability to monitor clinical responses would prompt earlier consideration of ET.

#### Case 3 – Treatment of Distal symptomatic vasospasm

This scenario consists of an unconscious patient with distal and diffuse vasospasm. This case aimed to assess: 1) whether a trial of induced hypertension is pursued prior to ET and 2) whether intra-arterial spasmolysis would be preferred as the first-line endovascular treatment.

#### Case 4 – Treatment of Proximal symptomatic vasospasm

A patient refractory to induced hypertension, now presents with proximal vasospasm involving the middle cerebral artery. The objective was to determine if the proximal location would shift decision-making toward angioplasty rather than spasmolysis.

### Data handling and analysis

Data were exported from the Survey Planet platform in CSV format and subsequently imported into R (version 4.5.0; www.r-project.org**)** for data processing and visualization using RStudio (version 2025.05.5 + 496). The analysis was primarily descriptive, focusing on the calculation of absolute frequencies and proportions (%) for nominal data. Data visualization was performed using bar charts, grouped bar chart, and pie (donut) charts to represent proportional data.

## Results

### Participants

**Fig. 1:**
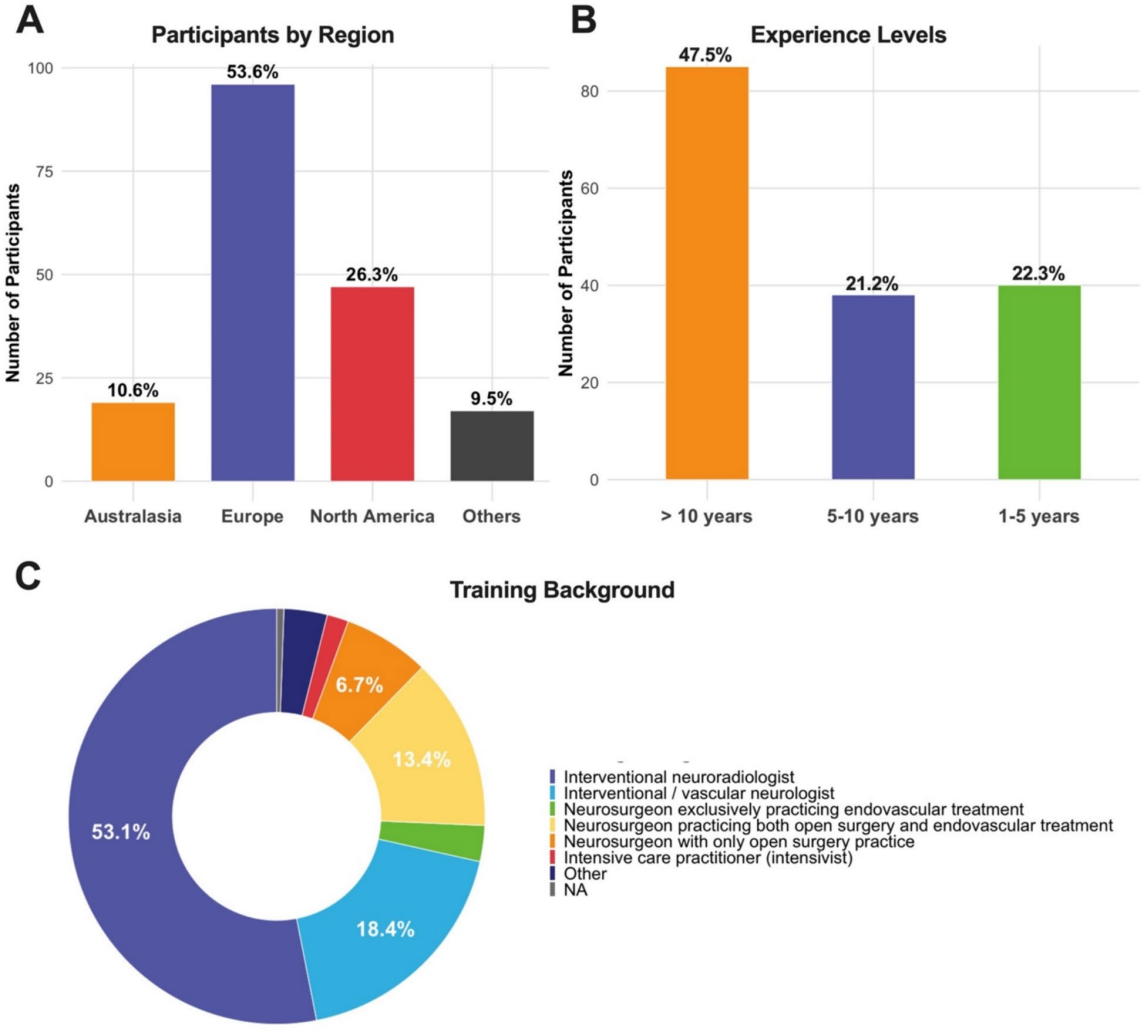
Bar and pie chart overview of proportion of respondents per (**A**) geographical region, (**B**) level of experience and (**C**) training background. In panel **A** and **B**, the y-axes represent absolute counts and the percentages the relative proportions. In panel C, for spacing reasons, only percentages above 5% are displayed. NA, not applicable

The study recruited 179 participants from 38 countries, with 19 participants (10.6%) from Australasia (including India and Japan), 47 (26.3%) from North America and 96 (53.6%) from Europe (see Fig. 1A). A total of 47.4% (85/179) of respondents had more than 10 years of experience, 21.2% (38/179) had 5 to 10 years, and 22.3% (40/179) had 1 to 5 years (Fig. 1B). The majority of respondents identified as interventional neuroradiologists (53.1%, 95/179), followed by interventional/vascular neurologists (18.4%, 33/179) and neurosurgeons practicing both open and endovascular surgery (13.4%, 24/179) (Fig. 1C). Most participants held senior positions, with 36.9% (66/179) identifying as consultants, 26.8% (48/179) as attending physicians and 12.3% (22/179) were chair of their respective departments.

### DCI diagnosis and first-line treatment

Diagnostic approaches to detect DCI varied among the respondents, reflecting differences in available resources and institutional protocols. In awake patients, the most common diagnostic approach combined clinical examination and transcranial Doppler sonography (TCD), as reported by 73 participants (41.0%). An additional 58 respondents (32.4%) indicated that they utilize a combination of clinical assessment, TCD, and CT imaging, including CT angiography (CTA), while 20 participants (11.2%) relied on clinical examination and CT perfusion. For unconscious SAH patients, diagnostic strategies were more diverse. TCD was the most widely used modality, reported by 132 participants (73.7%). CT imaging, including CTA and CT perfusion, was also frequently employed, with 87 respondents (48.6%) using CTA and 76 (42.5%) employing CTP. Repeated wake-up exams were applied by 65 respondents (36.3%), while 24 participants (13.4%) reported the use of multimodal monitoring, including brain tissue oxygen monitoring and cerebral microdialysis. Routinely scheduled conventional angiography was reported as a standard diagnostic measure by 26 respondents (14.4%).

Use of prophylactic nimodipine was common, although application forms varied, with 77 respondents (43%) administering it orally or intravenously, depending on clinical circumstances. Grounded nimodipine delivered via a gastric tube was applied by 73 participants (40.8%), while 26 respondents (14.5%) reported using intravenous nimodipine as the primary route.

Induced hypertension as a first-line treatment for symptomatic vasospasm or DCI was reported in 118 respondents (65.9%), though specific clinical criteria for its use were not uniformly defined. An additional 95 participants (53.1%) stated that the decision to implement induced hypertension depended on specific clinical scenarios. Notably, 41 respondents (22.9%) indicated that induced hypertension was not applied at their institution.

For those not applying induced hypertension, 28 participants (15.6%) cited insufficient clinical evidence as the primary reason, while 24 respondents (13.4%) expressed concerns regarding the risk of complications, such as pulmonary edema or cardiac decompensation. Negative results from existing studies, such as the Hypertension Induction in the Management of AneurysmaL subArachnoid haemorrhage with secondary IschaemiA (HIMALAIA) trial [19], were reported as influential by 12 respondents (6.7%).

### Endovascular treatment

**Fig. 2:**
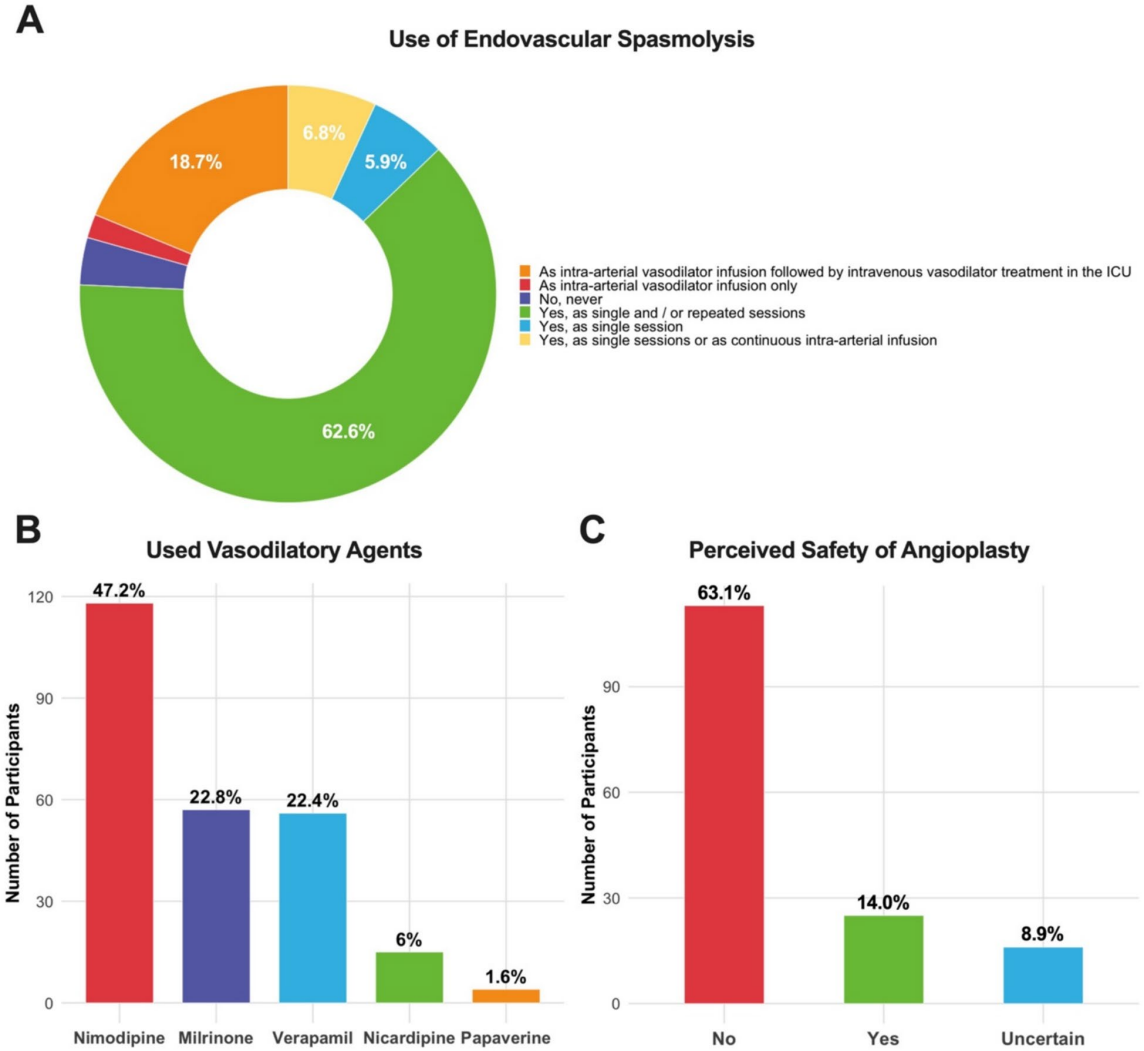
Pie and bar chart overview of absolute numbers and proportions (%) of respondents (**A**) modes of applying endovascular treatment, (**B**) vasodilatory agents used and (**C**) the perceived safety of angioplasty compared to intra-arterial spasmolysis. In plot **C**, No and Yes represent answers to the question do you assess angioplasty to be as safe as intra-arterial spasmolysis. In panel **B** and **C**, the x-axes represent absolute counts and the percentages the relative proportions. In plot **C**, for spacing reasons, only percentages above 5% are displayed

**Fig. 3:**
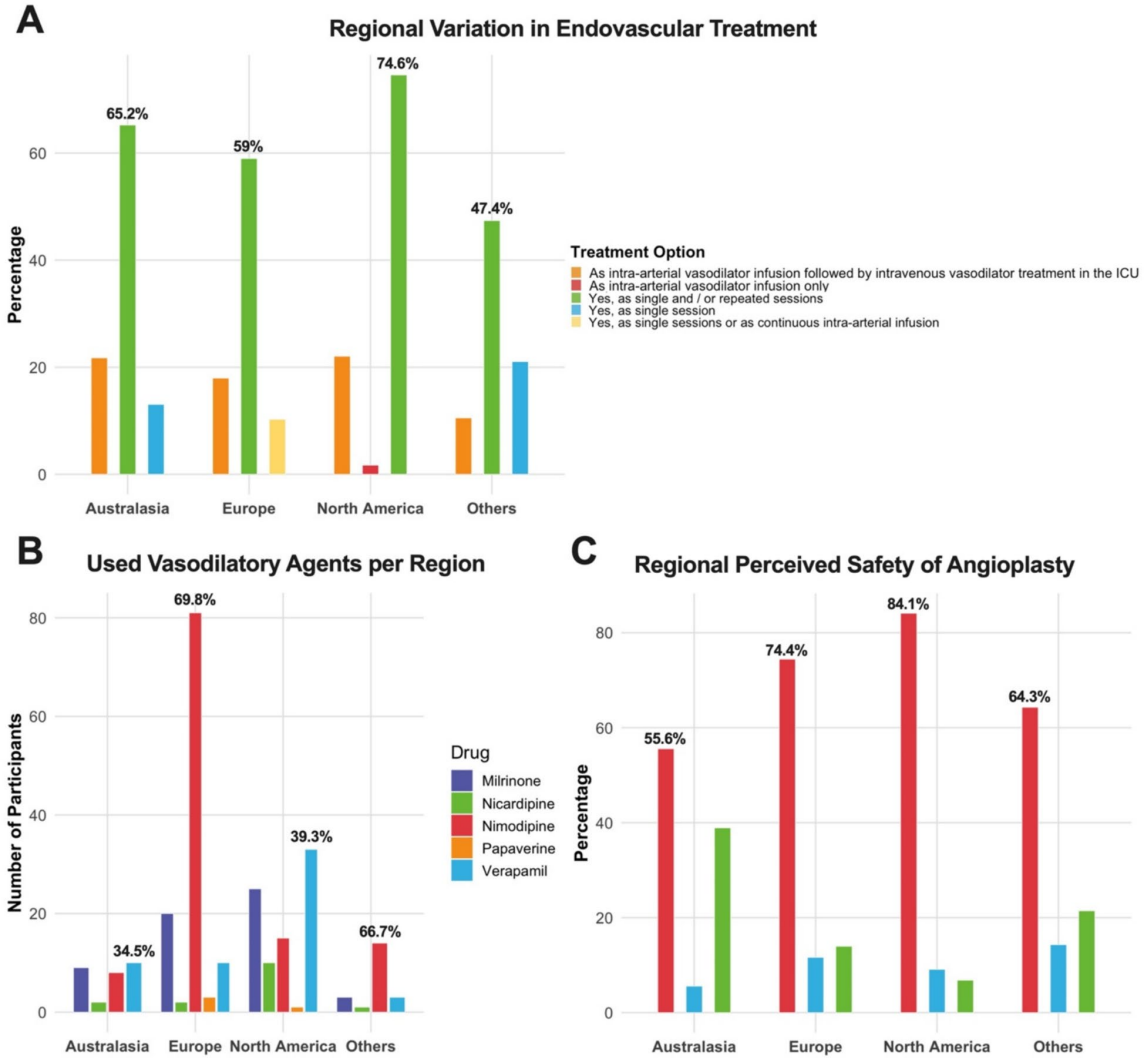
Grouped bar charts providing an overview of absolute numbers and proportions (%) of respondents per geographical region and (**A**) used modes of applying endovascular treatment, (**B**) vasodilatory agents used and (**C**) the perceived safety of angioplasty compared to intra-arterial spasmolysis. In plot **C**, No and Yes represent answers to the question do you assess angioplasty to be as safe as intra-arterial spasmolysis. In panel **A** and **C**, the x-axes represent absolute counts and the percentages the relative proportions. In panel **B**, the x-axis represents the absolute numbers of participants to simultaneously visualize the discrepancy in number of responders per geographical region

The application of ET was reported by the majority of participants. Of all respondents, 137 (76.5%) indicated that ET is applied as either single or repeated sessions, while an additional 15 participants (8.4%) reported that it is performed as either single session or as continuous intra-arterial infusions. Continuous intra-arterial vasodilator infusion followed by intravenous vasodilator treatment in the (ICU) care unit was described by 41 respondents (22.9%). The distribution of modes of applying ET is illustrated in Fig. 2A and compared between geographical regions in Fig. 3A. As multiple answers were possible for this question, the denominator for the proportions in the pie chart (Fig. 2A), was the total number of responses.

The requirement for a prior trial and failure of induced hypertension before initiating endovascular treatment varied among respondents. Of the 170 who answered this question, 61 (35.9%) indicated that induced hypertension is a prerequisite for endovascular intervention, whereas 89 participants (52.4%) reported that endovascular treatment can be initiated without a prior trial of induced hypertension.

Nimodipine was the most commonly reported intra-arterial vasodilatory agent, used by 118 respondents (65.9%). Milrinone and verapamil were also frequently employed, with 57 (31.8%) and 56 (31.2%) participants, respectively, indicating their use. Nicardipine was reported by 15 respondents (8.4%), and papaverine by four (2.2%). As multiple responses to this question were possible, results are plotted in a bar chart with the total number of responses in the denominator of the proportion (see Fig. 2B). Nimodipine was first choice among European respondents but not available as an infusion in other areas of the world. Verapamil was the favored vasodilator in North American respondents (see Fig. 3B). Contraindications for endovascular treatment were diverse. The most frequently cited contraindication was the presence of infarcted brain tissue and the associated risk of hemorrhagic transformation, reported by 47 respondents (26.3%). Cerebral edema was considered a contraindication by 31 participants (17.3%), and high vasopressor requirements by 20 respondents (11.2%). Sixteen respondents (8.9%) indicated that inadequate primary treatment by induced hypertension was a contraindication.

The primary modality used to assess treatment success or failure following endovascular intervention also varied. Clinical examination was the most common approach, employed by 64 respondents (35.8%). TCD was used by 40 respondents (23.3%), and follow-up catheter angiography by 27 (15.1%). Imaging modalities such as CTP and native CT were employed by 10 (5.6%) and 5 (2.8%) participants, respectively.

### Indications, safety, and complications of endovascular rescue treatment

Survey participants were queried regarding key indicators for the application of balloon angioplasty in the management of cerebral vasospasm. Among the 179 respondents, the most frequently cited indication was the failure of vasospasm resolution after vasodilator instillation, reported by 111 participants (62%). The anatomical location of the spastic segment was also considered highly relevant by 98 respondents (54.7%), while the presence of neurological symptoms was indicated by 62 participants (34.6%). The length of the spastic segment and the degree of vessel occlusion were considered important by 56 (31.8%) and 60 (33.5%) respondents, respectively. A smaller subset (15 respondents, 8.4%) provided other, miscellaneous indications for considering balloon angioplasty.

When asked about diagnostic modalities used to guide the discontinuation of endovascular spasmolysis after perceived treatment success, 103 participants (57.5%) reported relying primarily on clinical examination. TCD was also widely used, indicated by 88 respondents (49.2%). Angiographic resolution of vasospasm was considered a key indicator for cessation of treatment by 64 respondents (35.8%), while 43 participants (24%) utilized perfusion CT imaging.

The majority of respondents (113, 63.1%) considered balloon angioplasty to be more dangerous than pharmacological spasmolysis (Fig. 2C). In contrast, 25 participants (14%) regarded it as equally safe, and 16 respondents (8.9%) expressed uncertainty. This tendency was similar when comparing different geographical regions (see Fig. 3C).

While 87 respondents (48.6%) indicated that rising vasopressor requirements would not prompt discontinuation of spasmolysis, 36 participants (20.1%) considered it a valid indication to reduce or stop intra-arterial therapy. An additional 28 respondents (15.6%) stated that the decision would depend on specific clinical scenarios, underscoring the variability in institutional practices regarding this aspect of treatment.

Perceived complication rates associated with endovascular management of vasospasm were diverse. Severe increase in vasopressor demand was reported as the most commonly encountered complication, cited by 32 respondents (17.9%). Thromboembolic events, including iatrogenic stroke, were reported by 30 participants (16.8%), while vessel dissection was indicated by 28 respondents (15.6%). Hemorrhagic complications were noted by 22 participants (12.9%), and access site complications by 26 respondents (14.5%).

### Case scenarios

#### Case 1—ET as First- or second-line treatment (Conscious Patient)

In the scenario of a neurologically assessable patient with symptomatic DCI and a confirmed perfusion deficit and angiographic vasospasm, induced hypertension as a first-line intervention was only reported by 18 respondents (10.1%). The majority of 62 participants (34.6%) indicated that they would proceed with early pharmacological spasmolysis as the first-line treatment, whereas 18 respondents (10.1%) opted for balloon angioplasty as the initial approach. Notably, 50 participants (27.9%) indicated that balloon angioplasty would only be considered if there was no angiographic response to intra-arterial vasodilators.

#### Case 2—ET as First- or second-line treatment (Unconscious Patient)

If the same patient would be unconscious precluding neurological examination, 85 respondents (47.5%) reported proceeding with early endovascular spasmolysis as first-line intervention. This represents a marked increase compared to the conscious patient scenario in Case 1. Additionally, 27 participants (15.1%) selected balloon angioplasty as the initial treatment, and 26 respondents (14.5%) opted for induced hypertension.

#### Case 3—Treatment of Distal symptomatic vasospasm

In a patient with distal and diffuse vasospasm, single-session pharmacological spasmolysis was the most common response, reported by 45 participants (25.1%), while 25 respondents (14%) advocated for continuous intra-arterial spasmolysis that could be continued intra-arterially at the bedside. Balloon angioplasty was less frequently considered, selected by 11 respondents (6.1%), and 43 participants (24%) indicated that angioplasty would only be considered after failed spasmolysis.

#### Case 4—Treatment of proximal symptomatic vasospasm

In a patient with proximal vasospasm involving the middle cerebral artery, the most common response was to perform balloon angioplasty only after a failed response to intra-arterial vasodilators, selected by 56 participants (31.3%). An additional 28 respondents (15.6%) indicated that they would proceed directly with angioplasty as the first-line intervention. Continuous intra-arterial vasodilator infusion followed by intravenous vasodilator administration in the ICU was selected by 31 respondents (17.3%).

## Discussion

This international survey among neuro- care and neurointervention specialists highlights substantial variability in the use of endovascular treatment (ET) for delayed cerebral ischemia (DCI). Single or repeated intra-arterial pharmacological spasmolysis was the most commonly used strategy, employed as both first-line and rescue treatment. Early ET was frequently favored in unconscious patients, likely due to limited options for clinical monitoring. In cases of refractory proximal vasospasm, many centers adopted a stepwise approach, starting with vasodilator therapy and escalating to angioplasty only if needed. Despite its reported effectiveness, angioplasty remains associated with procedural risk concerns. These findings reveal a lack of consensus on indications and techniques, underlining the need for further research and standardized protocols to guide ET use in DCI.

A similar survey published in 2015, involving 344 respondents also reported significant variability in endovascular treatment strategies, with 91% of U.S. and 83% of non-U.S. respondents employing angioplasty for vasospasm management [16]. U.S. practitioners were more likely to use angioplasty for distal vasospasm (23% vs. 6%). The Mantra Study provided further questionnaire-based insights into the variability of endovascular treatment practice [20]. Among the 292 respondents in this survey, 81.2% reported using intra-arterial rescue procedures for DCI, with 68.6% employing a combination of vasodilators and angioplasty. Despite the widespread use of ET, angioplasty was less commonly applied as a standalone intervention. In a recent survey, 79% of respondents employed chemical vasodilators as the first-line treatment but only 17% considered them consistently effective, compared to 48% who perceived angioplasty as effective in over 75% of cases [17].

The current survey complements these findings by also inquiring about the use of continuous intra-arterial vasodilator infusion, a modality not addressed in the previous mentioned studies. Additionally, the inclusion of case-based clinical scenarios provides a nuanced perspective on treatment decision-making.

A future RCT should identify patients at high risk of infarction without ET, while considering treatment timing, the DCI definition, and criteria for first-line treatment failure. Such a trial could follow one of two approaches: a strict, protocol-driven algorithm applied uniformly across centers, or a pragmatic design allowing current practices, with ET used only in the randomization arm. The former would ensure standardization but may face challenges in recruitment and adherence. The latter could enable broader participation but may limit generalizability. Given the rarity of refractory DCI, successful implementation will require extensive multicenter collaboration to achieve adequate recruitment.

Although angiographic vasospasm is only one contributor to DCI, the two-hit hypothesis may offer a framework for understanding ET’s benefits. In the pre-injured brain, increased metabolic demand from cortical spreading depolarizations and neuroinflammation may heighten susceptibility to secondary insults like macrovasospasm. By resolving vasospasm angiographically, ET may help prevent irreversible infarction. This has, however, not been proven yet. Additionally, certain vasodilators, particularly nimodipine, may offer neuroprotection beyond macrovasospasm resolution, potentially mitigating microvasospasm and cerebral edema.

### Limitations

This study has several limitations to consider when interpreting the findings. First, the case-based scenarios were designed to elicit specific treatment strategies based on predefined clinical assumptions. While this structured approach aimed to capture targeted information, it may have inadvertently suggested preferred strategies, potentially influencing responses. An alternative design with a larger set of varied cases and open-ended responses could offer broader insights but might also reduce participation and complicate analysis. In addition, we did not include cases where the participants are queried how intensive care related complications such as pulmonary hypertension, neurogenic pulmonary edema, or ongoing vasopressor support (among many others) might have affected their treatment decisions.

Another limitation is the inability to calculate an exact response rate. The survey was distributed via multiple professional societies and online platforms without centralized tracking of email distribution. In addition, to cast a wider net, the link to the survey was postied on social media, impeding the retracement of response rate fully. While the survey aimed to reflect a broad range of clinical practices across regions and specialties, the sample should be regarded as a convenience sample, representing those who chose to participate.

## Conclusion

This international survey highlights substantial variability in the use of endovascular treatment (ET) for delayed DCI after aneurysmal subarachnoid hemorrhage. While single or repeated intra-arterial spasmolysis was the most common strategy, reported approaches varied notably with patient consciousness and vasospasm location. These findings underscore the need for consensus on optimal treatment algorithms, particularly for unconscious patients, where monitoring is limited, and for refractory proximal vasospasm, where angioplasty may be considered. Given limited clinical evidence and current heterogeneity, these insights offer important guidance for designing a future randomized controlled trial to establish standardized protocols for endovascular rescue therapy in DCI.

## Supplementary Information

Supplementary Material 1.

## Data Availability

The raw data of this analysis is available in the Supplemental Material as Appendix 1.

## Abbreviations

ET: Endovascular treatment
CT: Computed tomography
CTA: Computed tomography angiography
CTP: Perfusion computed tomography
DCI: Delayed cerebral ischemia
ICU: Intensive care unit
RCT: Randomized controlled trial
SAH: Aneurysmal subarachnoid hemorrhage
TCD: Transcranial Doppler
