# Supplementary material for: Endovascular treatment of delayed cerebral ischemia after aneurysmal subarachnoid hemorrhage – an international survey
Source: BMC Neurol. 2025 Oct 31;25:448. doi: 10.1186/s12883-025-04480-2 (PMC12579423; doi:10.1186/s12883-025-04480-2)
Supplement: Supplementary file 1 — Supplementary Material 1. [file 12883_2025_4480_MOESM1_ESM.docx]

**Supplemental Material**

**Appendix 1**

**Question Number of responses**

**Q1. In which country do you currently practice?**

- All 193 member states of the United Nations listed

**Q2. What description best fits your training background?**

- i) Interventional neuroradiologist 95
- ii) Interventional / vascular neurologist 33
- iii) Neurosurgeon exclusively practicing endovascular treatment 5
- iv) Neurosurgeon practicing both open surgery and endovascular treatment 24
- v) Neurosurgeon with only open surgery practice 12
- vi) Intensive care practitioner (intensivist) 6

**Q3. Approximately, how many years of experience in neuro-endovascular intervention do you have?**

- i) No direct experience (e.g., intensivist, neurologist etc.) 11
- ii) < 1 year 4
- iii) 1-5 years 40
- iv) 5-10 years 38
- v) > 10 years 85

**Q4. To have a rough estimate of your treatment experience, how many endovascular aneurysm treatments have you performed as the primary interventionalist during your career?**

- i) No direct experience (e.g., intensivist, neurologist etc.) 20
- ii) < 10 18
- iii) < 50 16
- iv) 50-100 17
- v) 100-200 21
- vi) > 200 33
- vii) > 500 53

**Q5. What is your current position?**

- i) resident 8
- ii) fellow 13
- iii) attending 48
- iv) consultant 66
- v) vice-chairman 14
- vi) chairman 22
- vii) Other – please specify 7

**Q6. How are awake SAH patients monitored for vasospasm and DCI at your institution?**

- i) Clinically 16
- ii) Clinically and transcranial Doppler sonography (TCD) 73
- iii) Clinically and CT imaging including CT perfusion 20
- iv) Clinically, TCD and CT imaging, including CT angiography (CTA) 58
- v) Other – please specify 10

**Q7. How are unconscious SAH patients monitored for vasospasms and DCI at your institution (a combination of multiple answers is possible)**

- i) Repeated wake-up exams 65
- ii) CT imaging – native 58
- iii) CT imaging – angiography 87
- iv) CT imaging – perfusion 76
- v) Routine angiogram 26
- vi) Transcranial doppler sonography (TCD) 132
- vii) Multimodal monitoring (i.e. brain tissue oxygen monitoring, cerebral microdialysis) 24
- viii) Other – please specify 7

**Q8. How is prophylactic nimodipine applied in SAH patients at your institution?**

- i) Orally, if needed, grounded and delivered via a gastric tube 73
- ii) Orally, or if not possible, intravenously 77
- iii) Intravenously 26
- iv) Not applied 1

**Q9. Is vasopressor induced hypertension applied as a treatment strategy for symptomatic vasospasm / DCI at your institution.**

- i) No 41
- ii) If yes, for which patients? Please describe. 118
- iii) It depends. Please describe a clinical scenario for which this treatment would be considered. 95

**Q10. If induced hypertension is not applied in your institution, what is the main reason for this decision? (combination of answers possible)**

- i) Insufficient clinical evidence 28
- ii) Negative results of existing studies (e.g. HIMALAIA) 12
- iii) Risk of side effects (e.g. pulmonary edema, cardiac decompensation, etc.) 24
- iv) Lack of experience 13
- v) Other – please specify 27

**Q11. Apart from published guidelines, does a standard operating procedure (SOP) exist at your institution for the treatment of delayed cerebral ischemia?**

- i) Yes 95
- ii) No 82

**Q12. Is endovascular rescue treatment (intra-arterial spasmolysis and / or balloon-angioplasty) for cerebral vasospasm after SAH considered as a treatment option at your institution?**

- i) No, never 8
  - - ii) Yes, as single session 13
- iii) Yes, as single and / or repeated sessions 137
- iv) Yes, as single sessions or as continuous intra-arterial infusion 15
- v) As intra-arterial vasodilator infusion followed by intravenous vasodilator treatment in the intensive care unit. 41
- vi) As intra-arterial vasodilator infusion only (no intravenous vasodilator treatment in the ICU) 4

**Q13. Is a prior trial and failure of induced hypertension a prerequisite to initiate endovascular treatment of cerebral vasospasm (either spasmolysis, angioplasty or both), at your institution?**

- i) Yes 61
- ii) No 89
- iii) It depends. Please describe a clinical scenario for which endovascular rescue treatment would be considered. 21

**Q14. Which vasodilatory agent is mainly used for the treatment of vasospasm at your institution?**

- i) Not applicable 0
- ii) Milrinone 57
- iii) Nicardipine 15
- iv) Nimodipine 118
- v) Papaverine 4
- vi) Verapamil 56
- vii) Other – please specify 6

**Q15. What would be considered a contra-indication for endovascular treatment of cerebral vasospasm at your institution? (multiple answers are possible)**

- i) Not applicable 74
- ii) Inadequate primary treatment by means of induced hypertension 16
- iii) Presence of infarcted brain tissue (i.e. risk of hemorrhagic transformation) 47
- iv) High vasopressor requirements 20
- v) Cerebral edema 31
- vi) Other – please specify 25

**Q16. What primary diagnostic modality is used to provide feedback on treatment success (or failure) after initiation of endovascular treatment?**

- i) Not applicable 4
- ii) Patient's clinical exam 64
- iii) CT imaging – native 5
- iv) CT imaging – perfusion 10
- v) Follow-up catheter angiogram 27
- vi) Transcranial Doppler 40
- vii) Other – please specify 17

**Q17. What is the first-line treatment applied at your institution in the following scenario. A 41-year-old female patient, presented with WFNS Grade 3 (A) from a ruptured left side anterior choroidal aneurysm (B).**


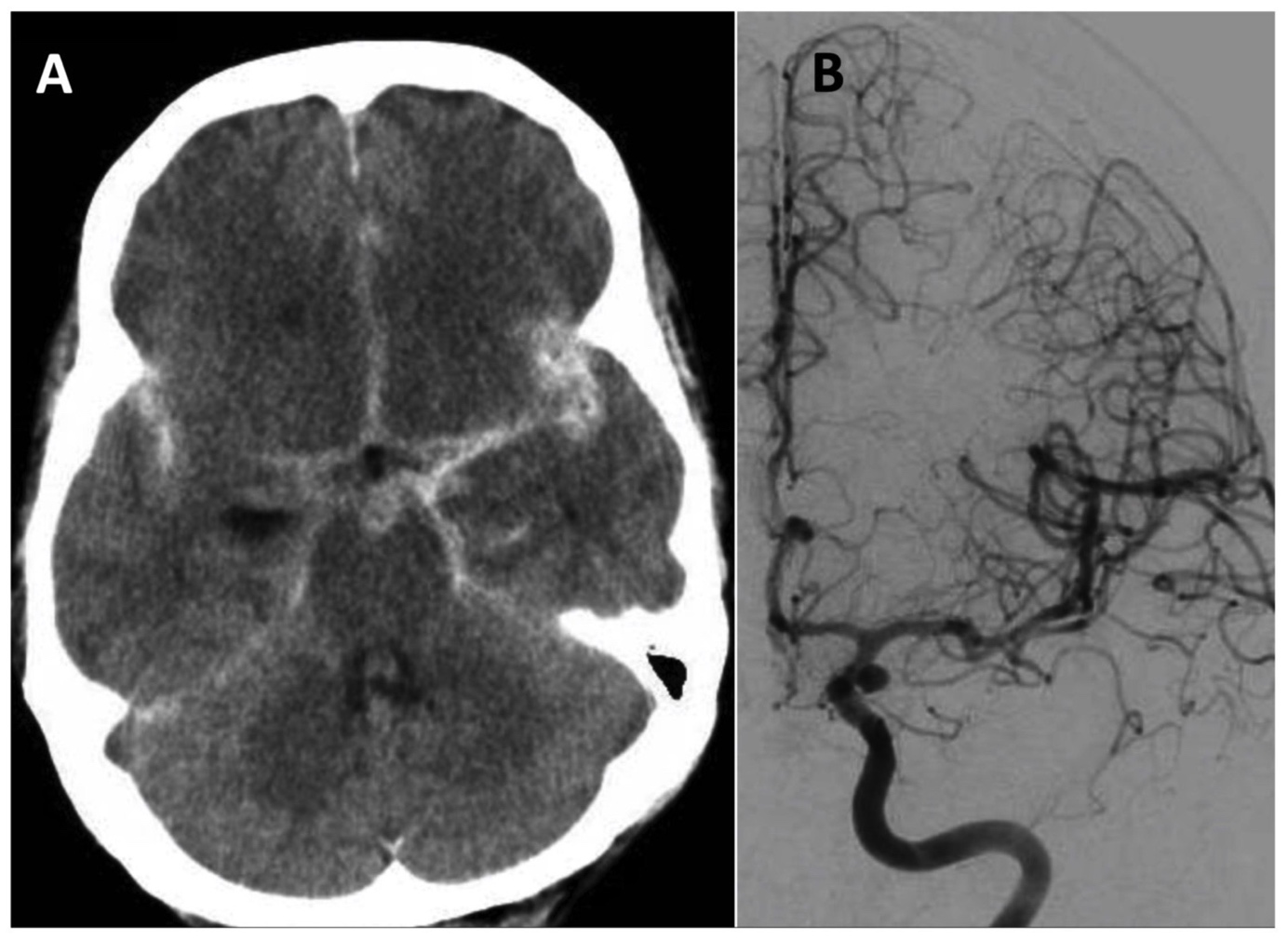
**After successful coiling, on day eight, she has right hemiparesis and dysphasia with perfusion CT scanning revealing a left side perfusion deficit in the middle cerebral artery territory. Angiography reveals vasospasm of the distal carotid, and proximal anterior and proximal and distal middle cerebral artery. Conventional CT did not demonstrate infarct.**
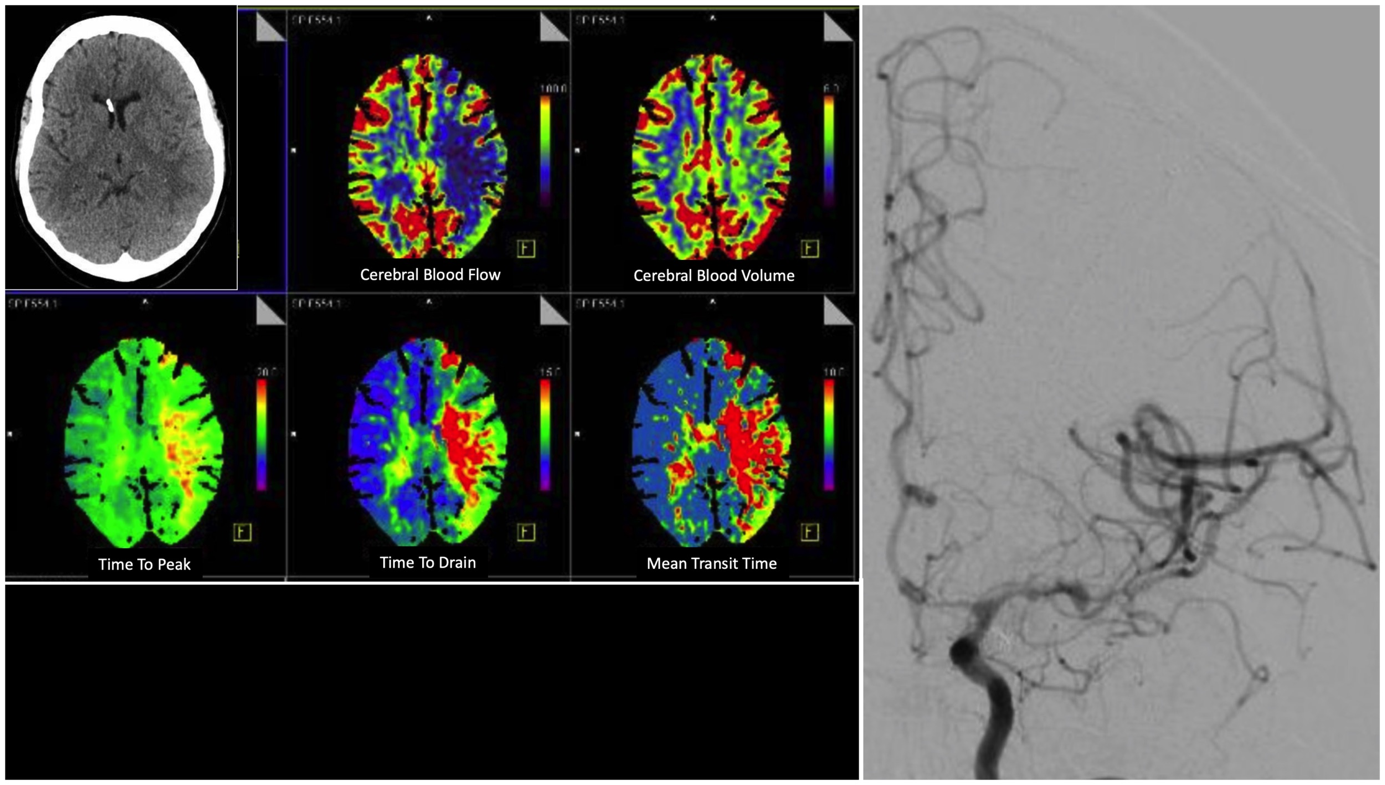


- i) Supportive treatment, normotension, normovolemia. 1
- ii) Supportive treatment, normovolemia and induced hypertension. 18
- iii) Supportive treatment, early endovascular spasmolysis 62
- iv) Balloon-angioplasty 18
- v) Balloon-angioplasty only if there is no angiographic response to an intra-arterial vasodilator 50
- vi) Other – please specify 19

**Q18. What would your first-line treatment be in the same scenario as above, given the fact that the patient is not neurologically assessable and the perfusion deficit (and angiographic vasospasm) was an incidental finding on routine scanning (and additional angiography) on day eight.**

- i) Supportive treatment, normotension, normovolemia. 9
- ii) Supportive treatment, normovolemia and induced hypertension. 26
- iii) Supportive treatment, early endovascular spasmolysis 85
- iv) Percutaneous transluminal angioplasty (PTA) 27
- v) Other – please specify 23

**Q19. What is the first-line treatment applied at your institution in the following case. A 41-year-old female presented with WFNS Grade 3 SAH (A), from a ruptured anterior communication artery aneurysm (B).**


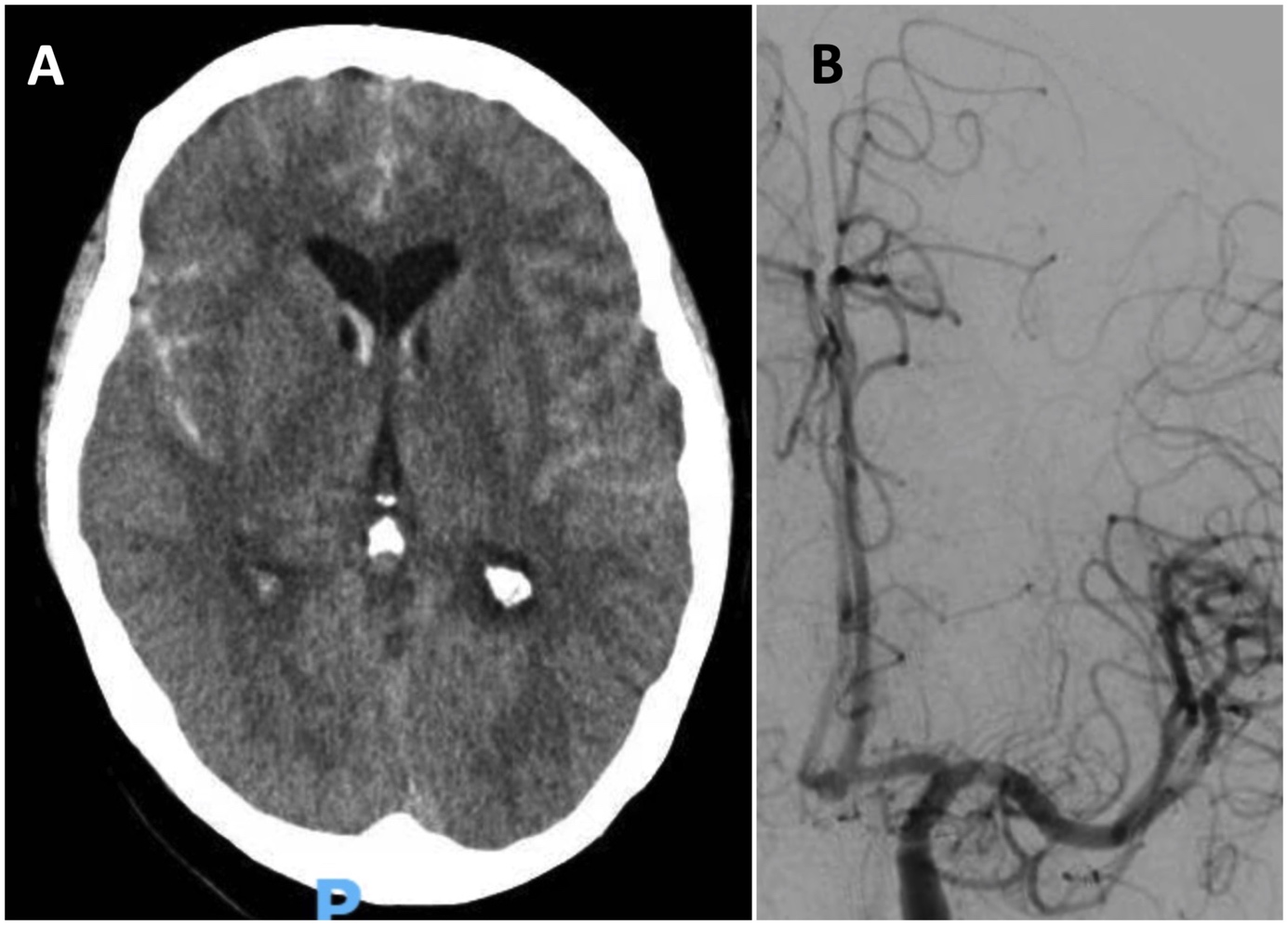
**After successful coiling, she has disorientation and a > 3 point drop in Glasgow coma scale on day eight, warranting intubation. Perfusion CT reveals bilateral perfusion deficits in both anterior cerebral artery territories. Angiography reveals bilateral vasospasm of the anterior cerebral arteries. CT did not demonstrate infarct.**


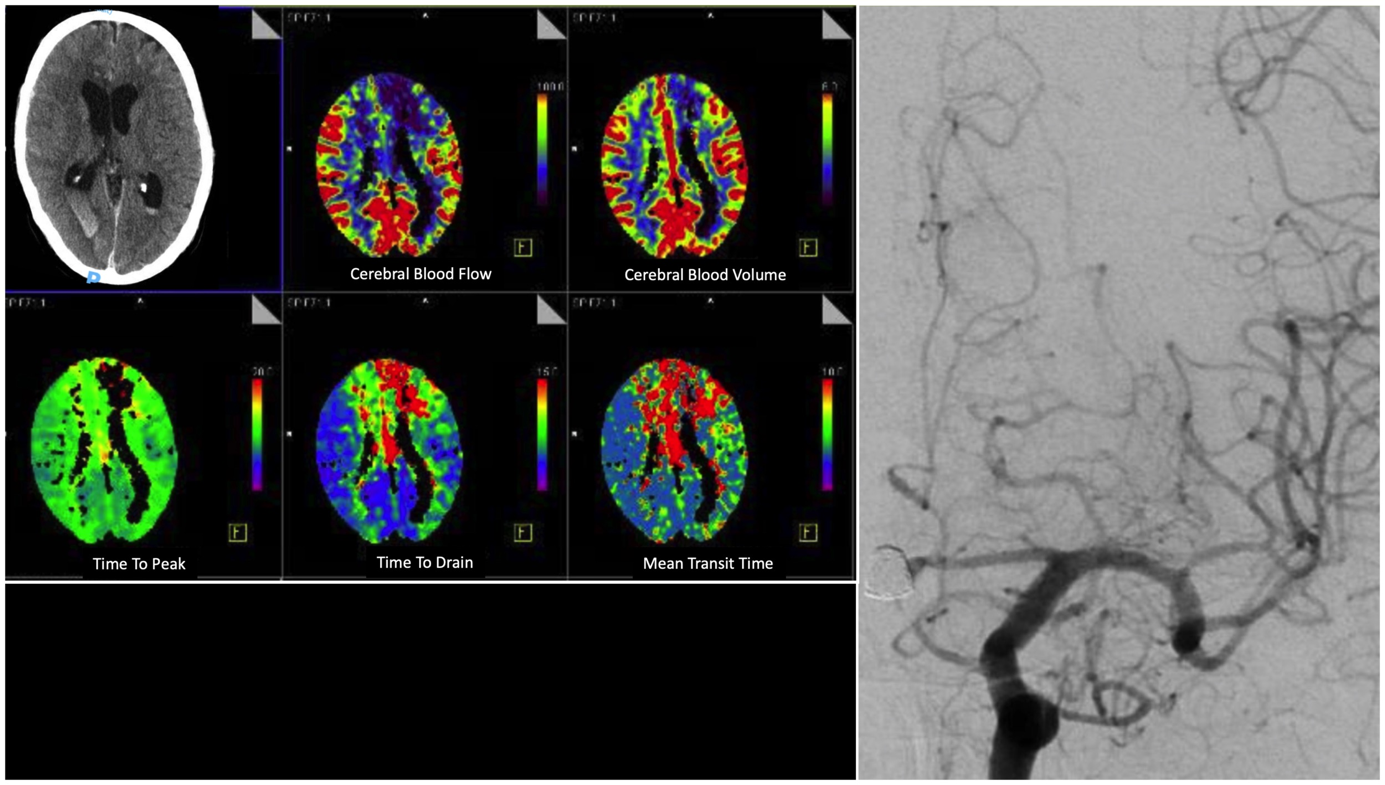


- i) Supportive treatment, normotension, normovolemia. 0
- ii) Supportive treatment, normovolemia and induced hypertension. 21
- iii) Single session spasmolysis 45
- iv) Continuous spasmolysis (continued bedside at the ICU) 25
- v) Balloon-angioplasty 11
- vi) Balloon-angioplasty only if there is no angiographic response to instillation with a vasodilatory agent 43
- vii) Other – please specify 24

**Q20. What is the first-line treatment applied at your institution in the following scenario. A 41-year-old female, presented with WFNS Grade 3 (A), from a ruptured middle cerebral artery aneurysm (B).**


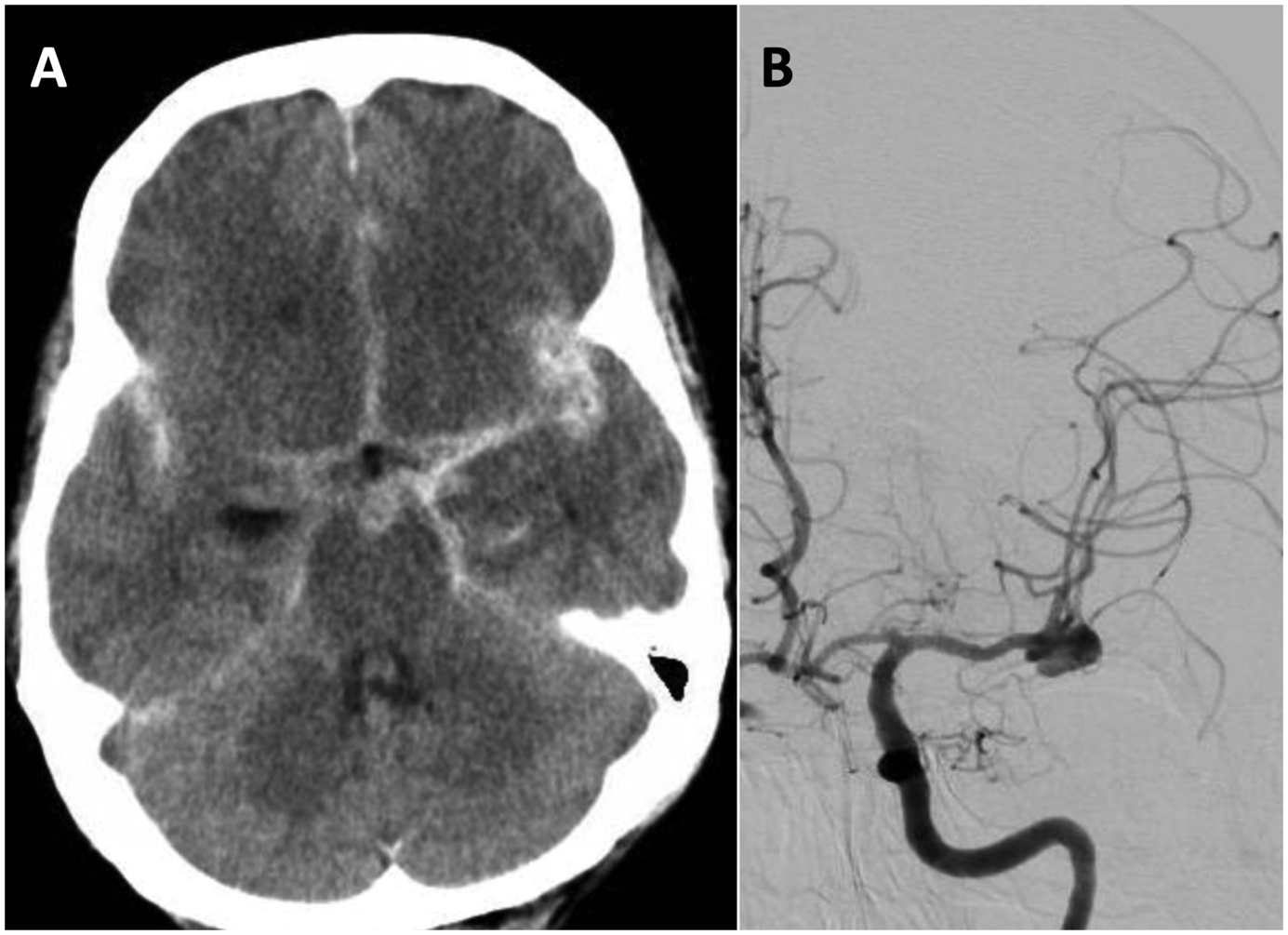
**After successful clipping (Day 1), she experiences right side hemiparesis and dysphasia not responsive to induced hypertension on Day 6. Perfusion CT reveals a left side perfusion deficit in the middle cerebral artery territory. Angiography reveals vasospasm of the proximal middle cerebral artery. Conventional CT did not demonstrate infarct.**


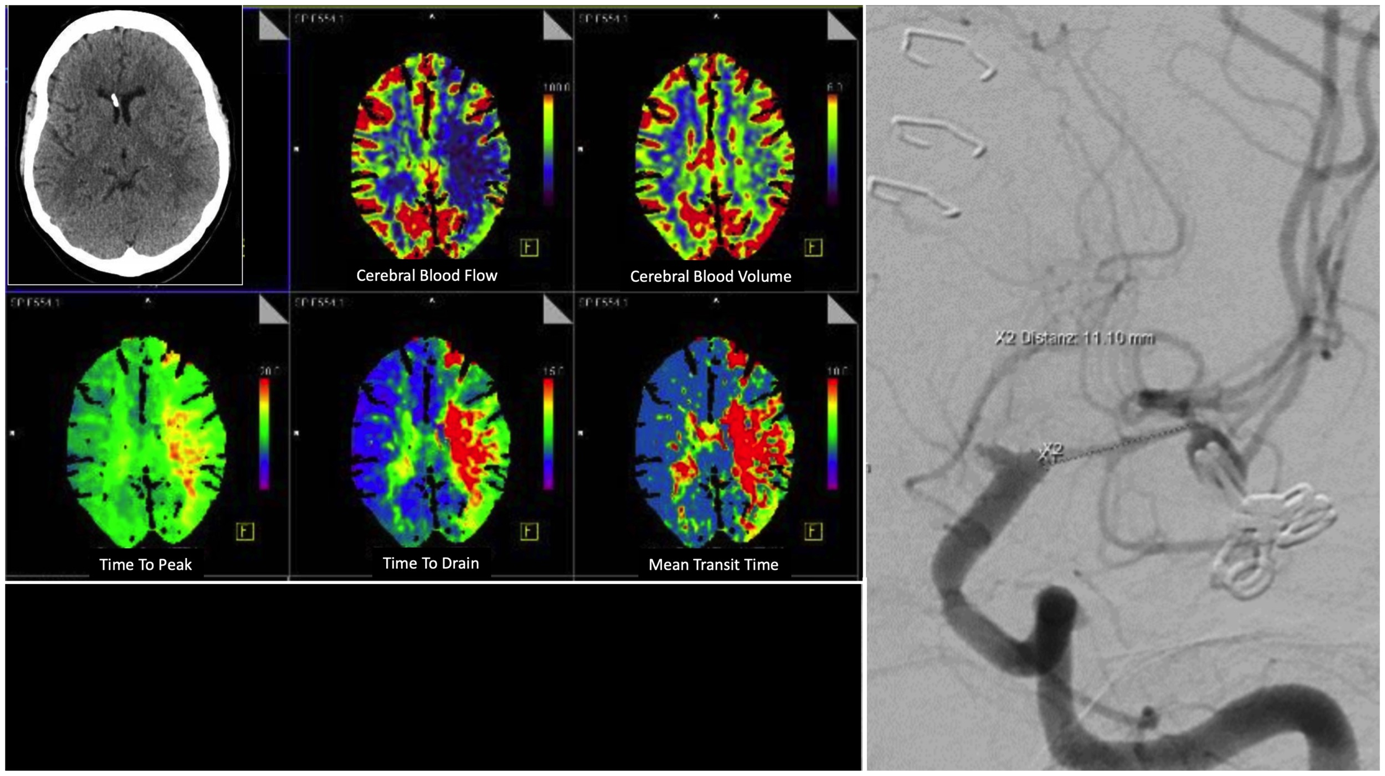


- i) Further supportive treatment, normovolemia and induced hypertension. 8
- ii) Single session IA vasodilator 22
- iii) IA vasodilator followed by IV continuous vasodilator 31
- iv) Balloon-angioplasty 28
- v) Balloon-angioplasty only if there is no angiographic response to an IA vasodilator 56
- vi) Other – please specify 20

**Q21. What is the most relevant indicator in favor of balloon-angioplasty (a combination of multiple answers is possible).**

- i) Not applicable 10
- ii) Patient is symptomatic 62
- iii) Failure of vasospasm resolution after vasodilator instillation 111
- iv) The location of the spastic segment 98
- v) The length of the spastic segment 56
- vi) The degree of vessel occlusion along the spastic segment 60
- vii) Other – please specify 15

**Q22. After successful endovascular treatment, what diagnostic modality is used at your institution to guide weaning / discontinuation of endovascular spasmolysis for cerebral vasospasm.**

- i) Not applicable 9
- ii) Clinical exam 103
- iiI) Angiographic improvement or resolution 64
- iv) Perfusion CT imaging 43
- v) Transcranial Doppler 88
- vi) Other – please specify 6

**Q23. If indicated appropriately, do you consider intra-arterial balloon angioplasty to be…**

- i) as safe as intra-arterial vasodilator application 25
- ii) more dangerous compared to intra-arterial vasodilator application 113
- iii) I have no clear opinion on the matter 16
- iv) Other – please specify 8

**Q24. Is rising vasopressor demand to maintain blood pressure (for DCI treatment, not in case or septic shock) an indication for you to reduce or stop endovascular spasmolysis for symptomatic cerebral vasospasm?**

- i) Not applicable 20
- ii) Yes 36
- iii) No 87
- iv) It depends. Please describe a clinical scenario for which you would consider premature discontinuation of endovascular spasmolysis. 28

**Q25. What would be the most common encountered complication related to endovascular management (both IA vasodilation and balloon-angioplasty) of DCI at your institution?**

- i) Not applicable 18
- ii) Thromboembolisms (i.e. iatrogenic stroke) 30
- iii) Vessel dissection 28
- iv) Severe increase in vasopressor demand 32
- v) Hemorrhagic complications 22
- vi) Access site complications 26
- vii) Other – please specify 12
